# The double burden of overnutrition and undernutrition in mother−child dyads in Kenya: demographic and health survey data, 2014

**DOI:** 10.1017/jns.2019.39

**Published:** 2020-01-24

**Authors:** Peninah Kinya Masibo, Felix Humwa, Teresia Njoki Macharia

**Affiliations:** 1 Global Programs for Research & Training, Kenya, Affiliate of the University of California San Francisco (UCSF), San Francisco, CA, USA; 2 Department of Nutrition, Moi University, School of Public Health, Nairobi, Kenya

**Keywords:** Undernutrition, Overnutrition, Mother−child pairs, Double burden, Kenya, Demographic health survey, aOR, adjusted OR, DHS, Demographic and Health Survey, KDHS, Kenya Demographic and Health Survey

## Abstract

The double burden of overnutrition and undernutrition is rapidly becoming a public health concern in low- and middle-income countries. We explored the occurrence of mother−child pairs of over- and undernutrition and the contributing factors using the 2014 Kenya Demographic and Health Survey data. A weighted sample of 7830 mother−child pairs was analysed. The children's nutritional status was determined using the WHO 2006 reference standards while maternal nutritional status was determined with BMI. Descriptive statistics, bivariate and multivariate logistic regression analysis were conducted. The proportion of overweight and obese mothers was 26 % (18·8 % overweight and 7·2 % obese). The prevalence of child stunting, underweight and wasting was 26·3, 12·8 and 5·1 %, respectively. Out of the overweight/obese mothers (weighted *n* 2034), 20 % had stunted children, 5·4 % underweight children and 3·1 % wasted children. Overweight/obese mother−stunted child pairs and overweight/obese mother−underweight child pairs were less likely to occur in the rural areas (adjusted OR (aOR) = 0·43; *P* < 0·01) in comparison with those residing in the urban areas (aOR = 0·54; *P* = 0·01). Children aged more than 6 months were more likely to be in the double burden dyads compared with children below 6 months of age (*P* < 0·01). The double burden mother−child dyads were more likely to be observed in wealthier households. Mother−child double burden is a notable public health problem in Kenya. Household wealth and urban residence are determinants of the double burden. There is need for target-specific interventions to simultaneously address child undernutrition and maternal overweight/obesity.

A double burden of malnutrition is the coexistence of undernutrition and overnutrition in the same settings either at the individual, household or community level^(1)^. At the individual level, the usual phenotype of the double burden is a stunted child who is also overweight or obese^(1)^. A household-level double burden is the co-existence of at least one member of a household with undernutrition and at least one member with overnutrition while a community-level double burden is when the two ends of the malnutrition spectrum are experienced in the same community, particularly among children^(2)^. The co-existence of over- and undernutrition within the same household is a complex challenge because of the shared household micro-environment. It would be expected that members of the same household have access to analogous foods and that dietary intake at the household level would result in alike nutrition outcomes. The quality of diet at the household level determines the nutritional status outcomes of the household members. Habitual consumption of energy-dense foods that are poor in nutrients contribute to adult overweight/obesity whilst they deprive young children of essential nutrients that they need for satisfactory growth. In addition, unequal intra-household food distribution may result in the mothers receiving a higher food ration in comparison with their children who receive less^(3)^.

The double burden phenomenon has been reported in low- and middle-income countries^(1,2,4–7)^. Earlier studies in sub-Saharan Africa demonstrate low double burden rates in households. Garret & Ruel^(8)^ reported < 10 % prevalence of double burden households in North Africa and sub-Saharan Africa. More recent studies have reported a higher prevalence of double burden mother−child pairs in specific areas of the population. For instance, a study in a large urban informal settlement in Nairobi, Kenya reported up to 40 % prevalence of stunting among children whose mothers were overweight and obese^(6)^. Although there has been a notable decline in childhood undernutrition levels in Kenya^(9,10)^, stunting in children remains higher than the WHO cut-off for public health significance of < 20 %^(11)^. On the other hand, the prevalence of obesity and overweight (BMI > 25 kg/m^2^) in Kenyan women is steadily increasing from 23 % in 2003^(12)^ to 25 % in 2008–2009^(13)^ and 38 % in 2014^(10)^. Fig. 1 demonstrates a conceptual framework for the double burden of over- and undernutrition adapted from the WHO Regional Office for South-East Asia^(14)^ based on the UNICEF 1997 causes of malnutrition^(15)^. Our framework is also informed by other authors^(4,16–19)^. This framework gives a background that underpins the causes of double burden of mother−child pairs of over- and undernutrition. The underlying determinants are of interest because a mother and her child's nutrition are closely related. Garret & Ruel^(8)^ hypothesised that in countries experiencing the nutrition transition, families may be able to purchase food but not in the right nutrient balance^(8)^. Affordable foods may be those that contain high energy with low concentration of other nutrients. On one hand, overweight and obesity among women results from a low-quality diet which is characterised by processed energy-dense foods with high sugar, salt and saturated fat and often low in fibre^(20,21)^. On the other hand, inadequate care, poor-quality diets and inadequate access to clean water and sanitation underpin the suboptimal nutrition outcomes in the children^(22)^. Micronutrient deficiencies in adults have also been associated with overweight and obesity^(23)^. In addition, a monotonous diet low in animal-source foods leads to micronutrient deficiencies which are associated with child stunting.

**Fig. 1.**
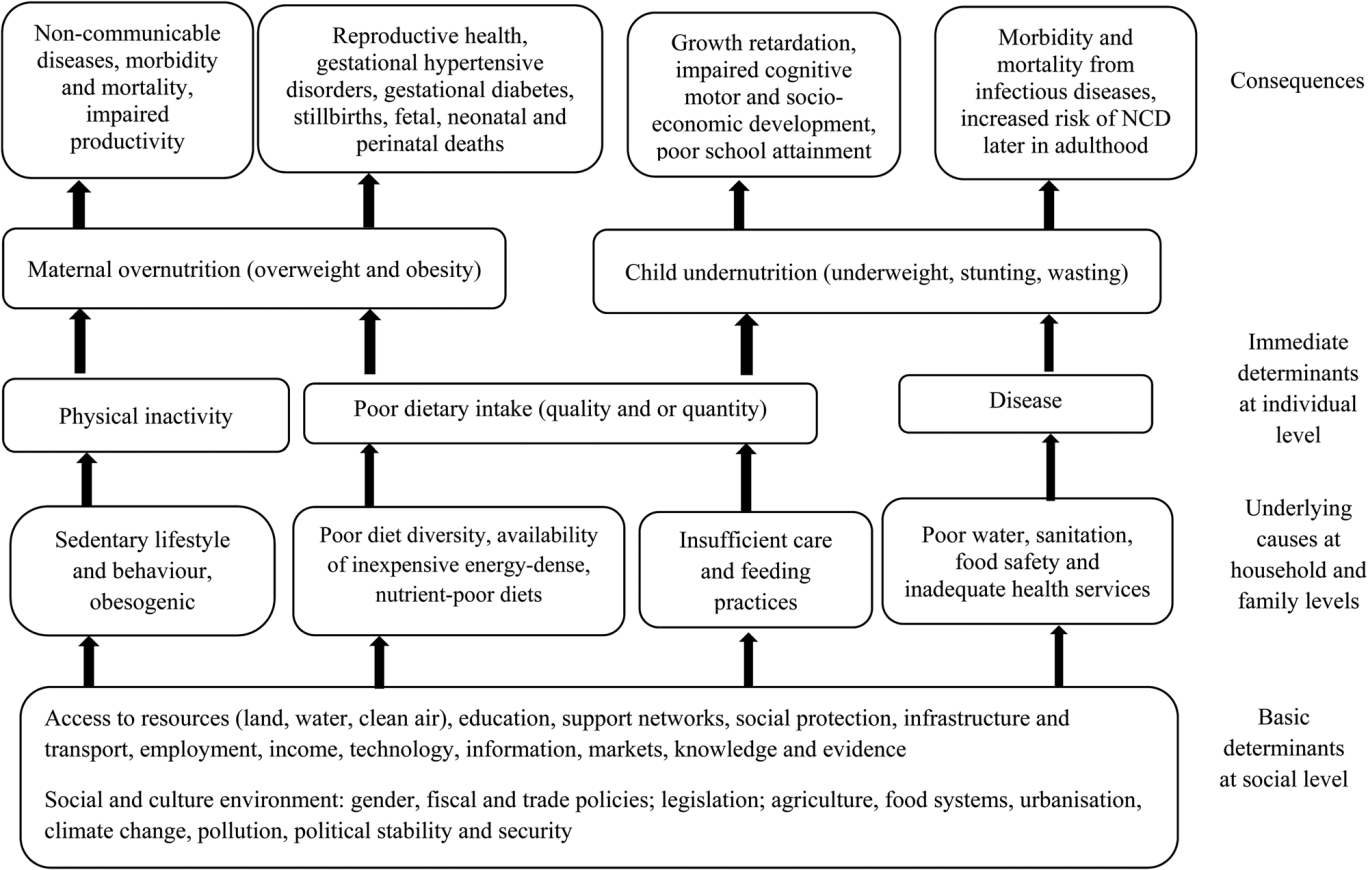
Conceptual framework for the determinants and outcomes of the dual burden of over- and undernutrition. NCD, non-communicable diseases. Source: adapted from the WHO^(14)^ and UNICEF^(15)^.

Undernutrition or overnutrition has health and development long-term consequences at individual and wider-society levels. Impaired cognitive development and poor school performance resulting from childhood undernutrition affect economic productivity in adult life^(16)^. Overweight and obesity lead to impaired economic productivity and increase expenditure on health care^(24)^. Both outcomes derail the public health care system and strain individual and household resources. It is important to quantify the double burden of malnutrition in Kenya to inform appropriate intervention strategies. Using the 2014 Kenya Demographic and Health Survey (KDHS) nationally representative population data, we present evidence on the magnitude and determinants of the mother−child pairs of double burden of malnutrition. The DHS is a high-quality standardised periodic survey providing data for a wide range of population, health and nutrition indicators. The DHS reports mainly present descriptive data and do not include detailed analysis associations or predictors of health and other developmental outcomes. In the present study, we conducted further analysis to present evidence on the magnitude and determinants of the mother−child pairs of double burden of malnutrition. A mother−child pair of double burden of malnutrition in this study was defined as an overweight or obese mother whose child is stunted, underweight or wasted.

## Methods

### Dataset

**Fig. 2.**
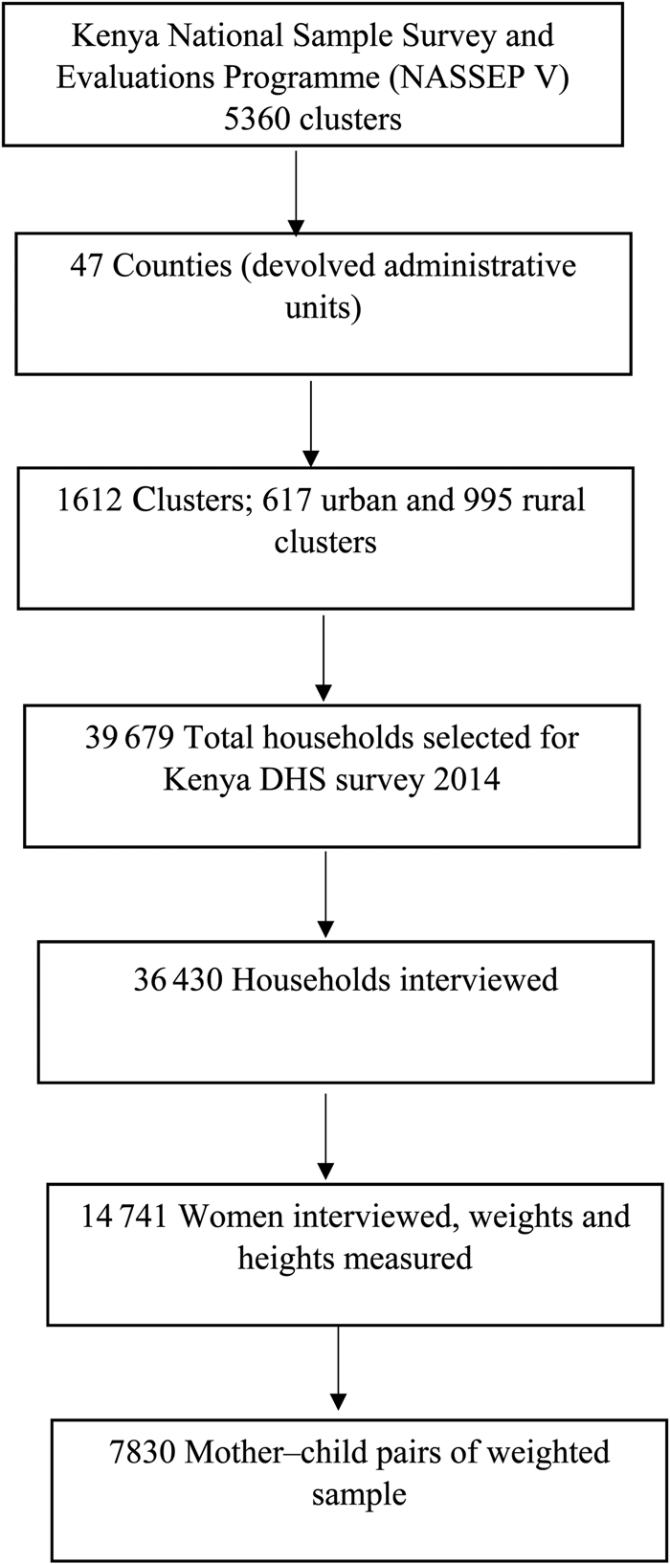
Flowchart describing the sampling and sample selection procedure for the analysis of the double burden of malnutrition in Kenya. DHS, Demographic and Health Survey.

The KDHS 2014 dataset was used for this analysis. The KDHS 2014 utilised multistage stratified cluster sampling methodology^(10)^. Samples of households within clusters (enumeration areas) were selected based on a master sampling frame of the Fifth National Sample Survey and Evaluations Programme (NASSEP V). In the NASSEP V, each of the forty-seven administrative counties in Kenya was stratified into rural and urban strata except for Nairobi and Mombasa counties that have only urban areas. Based on this sampling frame, a total of ninety-two sampling strata were utilised with 1612 clusters (Fig. 2). In the first level of selection all the 1612 clusters were included with equal probability from the NASSEP V frame. In the second stage of selection, a households listing was used as the sampling frame in which twenty-five households were selected from each cluster^(10)^. Residents in the selected households participated in the survey. Urban areas were oversampled, and the present analysis was based on weighted data to cater for the different sample proportions. A total of 39 679 households were selected for the 2014 survey and 36 430 were interviewed, yielding a 99 % response rate. A total of 14 741 women were interviewed in the households selected for the full household questionnaires which included measurements of height and weight for children less than 5 years and women. For our analysis, a weighted subsample of 7830 mother−child pairs was included based on the DHS children's recode data file^(25)^. The analysis included children under 5 years of age whose weight and height/length measurements were taken and whose mothers were interviewed and had their weights and heights measured. Sample weights were calculated based on the DHS sampling weights methodology^(26)^.

### Anthropometric measurements and nutritional status

In the KDHS, measurement of height and weight were taken for children born since January 2009 who were listed in the household questionnaire. Child weight was taken with electronic SECA digital scales to the nearest 100 g while child height was measured using Shorr height/length boards made by the Shorr productions^(10)^. The SECA digital weight scales used in the DHS survey were made with guidance from UNICEF^(10)^. Recumbent length was measured lying down for children younger than 24 months and for those with unknown age and less than 87 cm. Child nutritional status was determined using the WHO 2006 reference standard to calculate standard deviation *z*-scores. Childhood undernutrition was defined as height-for-age, weight-for-age and weight-for-height *z*-scores below −2 standard deviations from the WHO reference standard, being stunting, underweight and wasting, respectively. Childhood overweight was defined as weight-for-height >+2 standard deviation *z*-score. Children were excluded if their anthropometric measurements were invalid with height-for-age or weight-for-age *z*-scores below −6 standard deviation or above +6 standard deviation or with weight-for-height below −5 or above +5 standard deviations from the WHO reference standard^(27)^. Other child characteristics included in the analysis were age, sex, birth order and recent illness such as fever, diarrhoea or cough. Maternal weight and height were measured with SECA electronic scales made by the Shorr productions and Shorr height boards. BMI was derived as weight in kilograms divided by the square of height in metres.

Mothers' nutritional status was defined as: BMI <18·5 kg/m^2^, underweight; BMI 18·5–24·9 kg/m^2^, normal; BMI ≥25–29·9 kg/m^2^, overweight; BMI ≥30 kg/m^2^, obese. Pregnant women, women with a delivery within 2 months before the survey and those with invalid anthropometric measurements were excluded from this analysis. Mother−child pairs of double burden of over- and undernutrition was defined as an overweight or obese mother with a stunted, underweight or wasted child. Other maternal characteristics included in the analysis were age, maternal education, parity, marital status and work status.

### Household characteristics

Household wealth index, residence (rural or urban), source of drinking water and toilet facilities were included as independent characteristics in this analysis. The wealth index in the DHS is a proxy for household long-term living standards. The list of household assets and services used to calculate the wealth index in the DHS includes the type of materials used in house construction, water supply and sanitation facilities, electricity, radio, television, refrigerator, watch, type of vehicle, furniture, ownership of agriculture land and farm animals, cooking fuel, bank account, appliance items such as blender, water heater, camera or electronic generators^(28)^. The index is generated through principal components analysis that assigns a factor score for each asset. The resulting asset scores are standardised in a normal distribution^(29)^. The wealth index is grouped into five categories as poorest, poorer, middle, richer and richest households. Improved water sources are those adequately protected from contamination^(30)^. Improved water sources in the DHS include water from a piped water system, protected wells or springs, bottled or harvested rain water. Non-improved water sources are unprotected wells, springs, rivers, ponds, lakes and dams. Improved sanitation facilities were considered as those that separated human faecal matter from human contact hygienically^(30)^. In this study, improved sanitation facilities were flush toilets and ventilated and improved pit (VIP) latrines. Traditional pit latrines were categorised as non-improved sanitation facilities.

### Statistical analysis

Data were analysed using Stata software version 12.0 for Windows at the descriptive, bivariate and multivariate levels. Descriptive statistics reported frequencies and percentages for categorical data while means and standard errors were reported for continuous data. Sampling weights were applied to account for the survey sampling design used in the DHS. Bivariate logistic regression was used to check for relationships between the mother−child pairs and the household, maternal and child characteristics. Associations at bivariate and predictors at multivariate levels were reported in terms of OR and 95 % CI. Statistical significance was considered at *P* < 0·05.

## Results

We analysed a weighted total of 7830 mother−child pairs. The mothers were aged between 15 and 49 years and the children were under the age of 5 years. The mothers' mean age was 29·0 (95 % CI 28·7, 29·2) years. Slightly more than half of the mothers were aged between 20 and 30 years (53·3 %). A higher proportion resided in the rural areas (65·1 %) and had access to improved water sources (64·8 %) (Supplementary Table S1). The mothers’ mean BMI was 23·4 (95 % CI 23·2–23·6) kg/m^2^. The prevalence of overweight (BMI 25 to 29·9 kg/m^2^) among the mothers was 21·5 % while 8·4 % were obese (BMI ≥30 kg/m^2^) (Supplementary Table S1). The combined prevalence of overweight and obesity amongst the women was 26 %. The mean height-for-age, weight-for-age and weight-for-height *z*-scores among the children were −1·14, −0·63 and 0·02, respectively. About one-quarter (26·3 %) of the children were stunted, 12·8 % were underweight and 5·1 % were wasted. Less than 5 % (3·5 %) of children were overweight. Stunting levels were higher in boys (28·7 %) than girls (21·6 %). A quarter of the children had fever (25·1 %), 16·1 % had diarrhoea and 38·2 % had cough as a recent illness preceding the survey (Supplementary Table S1).

### Mother−child pairs of double burden of malnutrition

A mother−child pair of double burden was defined as an overweight or obese mother (BMI ≥ 25 kg/m^2^) with a stunted, underweight or wasted child. The weighted total number of mothers with overweight or obesity was 2034 (26·0 %). Out of these mothers, 571 had children who were either stunted, underweight or wasted distributed as follows: overweight/obese mother−stunted child pairs, 20 %; overweight/obese mother−underweight child pairs, 5·4 %; overweight/obese mother−wasted child, 3·1 % (Table 1). Overweight/obese mother−stunted child pairs occurred more in the urban areas (17·9 %) and among the richer (14·6 %) and richest (21·5 %) wealth quintile households. Similarly, the proportion of overweight/obese mother−stunted child pairs was higher among the mothers with post-secondary (college) level of education (15·17 %) (Table 1).

**Table 1.** Distribution of mother–child pairs of double burden of malnutrition by household, maternal and child characteristics, Kenya Demographic and Health Survey, 2014 (Numbers of pairs and percentages)

Background variable	Category	Overweight/obese mother–stunted child		Overweight/obese mother−underweight child		Overweight/obese mother−wasted child		Number of overweight/obese women (BMI ≥ 25 kg/m^2^)
		*n*	Weighted %	*n*	Weighted %	*n*	Weighted %	
Household characteristics								
Residence	Urban	177	17·93	52	5·4	30	2·8	1054
	Rural	221	7·85	58	1·72	33	0·78	980
Wealth quintile	Poorest	92	7·74	31	1·92	16	0·69	289
	Poorer	71	7·5	15	1·32	4	0·32	326
	Middle	68	7·8	22	2·82	16	1·92	362
	Richer	82	14·64	15	2·61	9	1·34	463
	Richest	85	21·52	27	7·26	18	3·81	594
Water source	Improved water source	243	12·4	75	3·51	52	1·97	1407
	Non-improved water source	145	9·23	33	1·96	10	0·51	568
Toilet facilities (*n* 392)	Improved toilet facilities	191	14·28	53	4·26	33	2·47	1140
	Non-improved toilet facilities	201	8·93	56	1·88	29	0·62	859
Maternal characteristics								
Age categories	<20 years	9	4·65	2	0·93	2	0·9	33
	20–30 years	177	9·87	44	2·15	29	1·16	918
	30–40 years	163	13·39	52	4·33	27	1·82	874
	40+ years	49	15·32	12	2·83	5	1·23	209
Education	None	55	7·75	26	3·06	19	1·31	208
	Completed primary	226	10·92	61	2·94	25	1·07	1031
	Completed secondary	86	11·92	16	1·98	16	1·91	542
	Post-secondary education	31	15·07	7	4·55	3	2·36	253
Marital status	Married	356	11·82	101	3·12	55	1·36	1798
	Single	42	7·68	9	1·37	8	1·34	236
Work status (*n* 395)	Working	268	11·24	43	2·9	31	1·32	590
	Not working	127	10·91	64	2·45	31	1·34	1432
Number of children ever born	1	47	7·53	12	2·33	12	1·81	293
	2–3	154	12·65	36	2·61	16	1·09	885
	4+	197	11·27	62	3·23	35	1·41	856
Child characteristics								
Age categories	< 6 months	8	2·02	3	0·21	8	0·92	131
	6–12 months	26	5·81	11	2·53	6	0·79	268
	13–24 months	94	14·51	22	3·04	19	2·3	366
	25–36 months	101	13·58	21	2·41	9	1·18	443
	37–59 months	169	12·81	53	3·69	21	1·25	826
Sex	Male	235	13·63	65	3·39	35	1·57	1029
	Female	163	8·65	45	2·24	28	1·14	1005
Birth order	1	66	7·62	18	2·06	13	1·34	391
	2–3	155	12·94	38	2·99	16	1·14	859
	4+	177	11·62	54	3·15	34	1·58	784
Recent illness								
Fever	No	303	11·68	83	3·02	43	1·44	1558
	Yes	95	9·66	27	2·29	20	1·15	474
	No	335	10·71	93	2·77	51	1·33	1749
Diarrhoea	Yes	63	13·28	17	3·1	12	1·53	284
	No	248	11·29	82	3·79	46	1·77	1260
Cough	Yes	150	10·89	28	1·32	17	0·73	772
Total		398	19·57	110	5·41	63	3·10	2034

### Mother−child pairs of double burden of malnutrition by household, maternal and child characteristics

#### Overweight/obese mother−stunted child pairs

Overweight/obese mother−stunted child pairs were less likely to occur in rural areas compared with the urban areas (adjusted OR (aOR) = 0·54; 95 % CI 0·38, 0·78; *P* = 0·001). Overweight/obese mother−stunted child pairs were less likely to occur in the poorest (aOR = 0·42; 95 % CI 0·22, 0·82; *P* = 0·01), poorer (aOR = 0·38; 95 % CI 0·21, 0·68; *P* < 0·001) and middle (aOR = 0·40; 95 % CI 0·23, 0·69; *P* < 0·001) wealth quintile households compared with the richest wealth quintile households. Overweight/obese mother−stunted child pairs were more likely to occur if the child was male (aOR = 0·57; 95 % CI 0·43, 0·76; *P* < 0·0001) compared with the mother−child pairs with female children. The risk of having an overweight/obese mother−stunted child pair was highest among the children aged 13–24 months (aOR = 6·81; 95 % CI 2·63, 17·64; *P* < 0·0001) compared with children less than 6 months old (Table 2).

**Table 2.** Predictors of overweight/obese mother−stunted child pairs of double burden of malnutrition by household, maternal and child characteristics, Kenya Demographic and Health Survey, 2014* (Unadjusted odds ratios (uOR), adjusted odds ratios (aOR) and 95 % confidence intervals)

Background variable	Category	Overweight/obese mother−stunted child					
		uOR	95 % CI	*P*	aOR	95 % CI	*P*
Household characteristics							
Residence	Urban	Reference			Reference		
	Rural	0·39	0·28, 0·54	<0·0001	0·54	0·38, 0·78	0·001
Wealth quintile	Poorest	0·31	0·18, 0·51	<0·0001	0·42	0·22, 0·82	0·01
	Poorer	0·30	0·18, 0·49	<0·0001	0·38	0·21, 0·68	0·001
	Middle	0·31	0·19, 0·50	<0·0001	0·40	0·23, 0·69	0·001
	Richer	0·63	0·37, 1·05	0·074	0·76	0·43, 1·32	0·328
	Richest	Reference			Reference		
Water source	Improved water source	Reference			Reference		
	Non-improved water source	0·72	0·52, 0·99	0·044	1·10	0·78, 1·56	0·583
Toilet facilities							
	Improved toilet facilities	Reference			Reference		
	Non-improved toilet facilities	0·59	0·43, 0·80	0·001	1·04	0·73, 1·48	0·837
Maternal characteristics							
Age categories							
	<20 years	0·27	0·11, 0·64	0·003	0·73	0·24, 2·20	0·577
	20–30	0·61	0·37, 0·98	0·04	0·66	0·36, 1·19	0·164
	30–40	0·85	0·53, 1·37	0·511	0·77	0·46, 1·27	0·305
	40+	Reference			Reference		
Education							
	None	0·47	0·25, 0·90	0·022	0·82	0·38, 1·78	0·632
	Completed primary	0·69	0·41, 1·16	0·162	1·15	0·61, 2·16	0·664
	Completed secondary	0·76	0·42, 1·40	0·381	0·96	0·49, 1·90	0·912
	Post-secondary education	Reference			Reference		
Marital status							
	Married	Reference			Reference		
	Single	0·62	0·40, 0·96	0·032	0·79	0·49, 1·28	0·344
Work status							
	Working	1·03	0·72, 1·48	0·855			
	Not working	Reference					
Number of children ever born							
	1	Reference			Reference		
	2–3	1·78	1·11, 2·85	0·016	1·01	0·40, 2·58	0·982
	4+	1·56	0·99, 2·45	0·053	0·81	0·24, 2·73	0·736
Child characteristics							
Age categories							
	<6 months	Reference			Reference		
	6–12 months	2·99	1·15, 7·79	0·025	2·66	0·99, 7·14	0·052
	13–24 months	8·23	3·23, 20·95	<0·0001	6·81	2·63, 17·64	<0·0001
	25–36 months	7·62	3·19, 18·21	<0·0001	5·84	2·36, 14·43	<0·0001
	37–59 months	7·12	2·98, 17·03	<0·0001	6·06	2·42, 15·16	<0·0001
Sex							
	Male	Reference			Reference		
	Female	0·60	0·46, 0·79	<0·0001	0·57	0·43, 0·76	<0·0001
Birth order							
	1	Reference			Reference		
	2–3	1·80	1·24, 2·62	0·002	1·82	0·87, 3·82	0·113
	4+	1·59	1·06, 2·40	0·025	2·50	0·87, 7·19	0·088
Recent illness							
Fever	No	Reference			Reference		
	Yes	0·81	0·59, 1·11	0·189	0·81	0·60, 1·11	0·188
Diarrhoea	No	Reference					
	Yes	1·27	0·81, 2·01	0·292			
Cough	No	Reference					
	Yes	0·96	0·70, 1·32	0·806			

* Bivariate logistic regression was used to report associations by use of uOR. Variables that had a *P* < 0·2 in the bivariate model were controlled for in the multivariate model (residence, wealth quintile, water source, toilet facilities, maternal age, maternal education, marital status, number of children, child age, child sex, child birth order, fever). Multivariate logistic regression was used to obtain the independent determinants and reported by use of aOR.

#### Overweight/obese mother−underweight child pairs

The odds of overweight/obese mother−underweight child pairs were lower among mother−child pairs living in rural areas than those in the urban areas (aOR = 0·43; 95 % CI 0·23, 0·79; *P* = 0·007). At the bivariate level of analysis, household wealth was significantly associated with overweight/obese mother−underweight child pairs. The poorest, poorer, middle and richer wealth quintiles had lower odds of observing overweight/obese mother−underweight child pairs (Table 3). However, the results were not statistically significant in the multivariate adjusted model. The risk of having an overweight/obese mother−underweight child pair increased with child's age with the highest odds observed in the 37- to 59-month-old compared with less than 6-month-old children (aOR = 15·04; 95 % CI 3·06, 73·84; *P* = 0·001). Reported childhood diarrhoea in the recent past was not significantly associated with overweight/obese mother−underweight child pairs. Overweight/obese mother−underweight child pairs were less likely if the child had reported cough in the recent past compared with the children without a cough (aOR = 0·36; 95 % CI 0·20, 0·64; *P* < 0·001) (Table 3).

**Table 3.** Predictors of overweight/obese mother−underweight child pairs of double burden of malnutrition by household, maternal and child characteristics, Kenya Demographic and Health Survey, 2014* (Unadjusted odds ratios (uOR), adjusted odds ratios (aOR) and 95 % confidence intervals)

Background variable	Category	Overweight/obese mother−underweight child					
		uOR	95 % CI	*P*	aOR	95 % CI	*P*
Household characteristics							
Residence	Urban	Reference			Reference		
	Rural	0·31	0·18, 0·52	<0·0001	0·43	0·23, 0·79	0·007
Wealth quintile	Poorest	0·25	0·12, 0·52	<0·0001	0·68	0·25, 1·87	0·46
	Poorer	0·17	0·07, 0·39	<0·0001	0·44	0·17, 1·14	0·09
	Middle	0·37	0·16, 0·84	0·017	0·84	0·33, 2·18	0·724
	Richer	0·34	0·14, 0·84	0·019	0·56	0·23, 1·40	0·213
	Richest	Reference			Reference		
Water source	Improved water source	Reference			Reference		
	Non-improved water source	0·55	0·32, 0·95	0·032	0·89	0·49, 1·61	0·7
Toilet facilities							
	Improved toilet facilities	Reference			Reference		
	Non-improved toilet facilities	0·43	0·26, 0·72	0·001	0·71	0·43, 1·17	0·182
Maternal characteristics							
Age categories							
	<20 years	0·32	0·06, 1·83	0·202	0·43	0·07, 2·65	0·36
	20–30 years	0·75	0·33, 1·71	0·497	0·55	0·24, 1·26	0·156
	30–40 years	1·55	0·69, 3·51	0·29	1·08	0·48, 2·40	0·857
	40+ years	Reference			Reference		
Education							
	None	0·66	0·20, 2·13	0·489			
	Completed primary	0·64	0·21, 1·91	0·419			
	Completed secondary	0·42	0·13, 1·41	0·162			
	Post-secondary education	Reference					
Marital status							
	Married	Reference					
	Single	0·43	0·18, 1·02	0·055			
Work status							
	Working	1·19	0·65, 2·177	0·578			
	Not working	Reference					
Number of children ever born							
	1	Reference					
	2–3	1·12	0·50, 2·52	0·779			
	4+	1·40	0·64, 3·08	0·403			
Child characteristics							
Age categories							
	< 6 months	Reference			Reference		
	6–12 months	12·07	2·15, 67·62	0·005	12·04	2·15, 67·30	0·005
	13–24 months	14·57	2·88, 73·83	0·001	13·99	2·69, 72·69	0·002
	25–36 months	11·48	2·27, 58·12	0·003	10·40	2·03, 53·19	0·005
	37–59 months	17·80	3·66, 86·53	<0·0001	15·04	3·06, 73·84	0·001
Sex							
	Male	Reference			Reference		
	Female	0·65	0·40, 1·07	0·091	0·68	0·42, 1·11	0·124
Birth order							
	1	Reference					
	2–3	1·47	0·70, 3·06	0·307			
	4+	1·55	0·74, 3·23	0·244			
Recent illness							
Fever	No	Reference					
	Yes	0·76	0·41, 1·39	0·367			
Diarrhoea	No	Reference					
	Yes	1·12	0·55, 2·30	0·753			
Cough	No	Reference			Reference		
	Yes	0·34	0·19, 0·60	<0·0001	0·36	0·20, 0·64	<0·0001

* Bivariate logistic regression was used to report associations by use of uOR. Variables that had a *P* < 0·2 in the bivariate model were controlled for in the multivariate model (residence, wealth quintile, water source, toilet facilities, cough, maternal age, child age and child sex). Multivariate logistic regression was used to obtain the independent determinants and reported by use of aOR.

#### Overweight/obese mother−wasted child pairs

Mother−child pairs with no access to an improved water source and improved toilet facilities were less likely to be an overweight/obese mother−wasted child pair compared with those having improved water sources and improved toilet facilities (aOR = 0·38; 95 % CI 0·16, 0·19; *P* = 0·027; and aOR = 0·37; 95 % CI 0·17, 0·79; *P* = 0·011, respectively; Table 4). At the bivariate level of analysis, rural residents were less likely to have overweight/obese mother−wasted child pairs compared with urban residents (uOR = 0·27; 95 % CI 0·14, 0·522; *P* < 0·0001). Living in the poorest (uOR = 0·17; 95 % CI 0·07, 0·422; *P* < 0·0001) and poorer (uOR = 0·08; 95 % CI 0·02, 0·26; *P* < 0·0001) wealth quintile households was less likely to be associated with overweight/obese mother−wasted child pairs compared with those living in the richest wealth quintile households. Overweight/obese mother−underweight child pairs were less likely if the child had reported a cough in the recent past compared with the children without a cough (aOR = 0·41; 95 % CI 0·19, 0·90; *P* = 0·025) (Table 4).

**Table 4.** Predictors of overweight/obese−wasted child pairs of double burden of malnutrition by household, maternal and child characteristics, Kenya Demographic and Health Survey, 2014* (Unadjusted odds ratios (uOR), adjusted odds ratios (aOR) and 95 % confidence intervals)

Background variable	Category	Overweight/obese mother−wasted child					
		uOR	95 % CI	*P*	aOR	95 % CI	*P*
Household characteristics							
Residence	Urban	Reference			Reference		
	Rural	0·27	0·14, 0·522	<0·0001	0·53	0·23, 1·26	0·152
Wealth quintile	Poorest	0·17	0·07, 0·422	<0·0001	0·83	0·22, 3·13	0·778
	Poorer	0·08	0·02, 0·26	<0·0001	0·27	0·07, 1·06	0·06
	Middle	0·49	0·19, 1·29	0·151	1·33	0·35, 5·01	0·677
	Richer	0·34	0·13, 0·93	0·036	0·49	0·17, 1·38	0·179
	Richest	Reference			Reference		
Water source	Improved water source	Reference			Reference		
	Non-improved water source	0·25	0·11, 0·58	0·001	0·38	0·16, 0·90	0·027
Toilet facilities							
	Improved toilet facilities	Reference			Reference		
	Non-improved toilet facilities	0·25	0·13, 0·48	<0·0001	0·37	0·17, 0·79	0·011
Maternal characteristics							
Age categories							
	<20 years	0·73	0·11, 4·87	0·749			
	20–30 years	0·94	0·29, 3·03	0·918			
	30–40 years	1·49	0·45, 4·94	0·509			
	40+ years	Reference					
Education							
	None	0·55	0·13, 2·38	0·423			
	Completed primary	0·45	0·11, 1·91	0·276			
	Completed secondary	0·81	0·19, 3·42	0·773			
	Post-secondary education	Reference					
Marital status							
	Married	Reference					
	Single	0·98	0·38, 2·50	0·965			
Work status							
	Working	0·99	0·49, 2·00	0·971			
	Not working	Reference					
Number of children ever born							
	1	Reference					
	2–3	0·60	0·23, 1·56	0·297			
	4+	0·78	0·33, 1·86	0·571			
Child characteristics							
Age categories							
	< 6 months	Reference					
	6–12 months	0·86	0·18, 4·07	0·85			
	13–24 months	2·54	0·88, 7·35	0·086			
	25–36 months	1·28	0·39, 4·21	0·679			
	37–59 months	1·37	0·45, 4·18	0·583			
Sex							
	Male	Reference					
	Female	0·72	0·36, 1·43	0·346			
Birth order							
	1	Reference					
	2–3	0·85	0·33, 2·19	0·739			
	4+	1·18	0·50, 2·82	0·703			
Recent illness							
Fever	No	Reference					
	Yes	0·80	0·39, 1·62	0·53			
Diarrhoea	No	Reference					
	Yes	1·16	0·48, 2·77	0·746			
Cough	No	Reference			Reference		
	Yes	0·41	0·19, 0·88	0·022	0·41	0·19, 0·90	0·025

* Bivariate logistic regression was used to report associations by use of uOR. Variables that had a *P* < 0·2 in the bivariate model were controlled for in the multivariate model (residence, wealth quintile, water source, toilet facilities, cough). Multivariate logistic regression was used to obtain the independent determinants and reported by use of aOR.

## Discussion

We found that one-quarter of Kenyan children less than 5 years old were stunted, one in every ten children was underweight and less than 5% of children were wasted. We found a lower prevalence of childhood undernutrition from what has been previously reported in Kenya^(10,12,13,31–33)^. It is important to note the downward trend of childhood stunting in Kenya from 40 % in 1993 to 35 % in 2008–20009^(10)^, and a further decline to 26 % in 2014^(10)^. Though the prevalence of childhood undernutrition is reducing in the country, the prevalence of adult overweight and obesity is steadily rising. Overweight and obesity among women of reproductive age increased from 23 % in 2003^(12)^ to 25 % in 2008–2009^(13)^, and 33 % in 2014^(10)^. In this study, we found that one-quarter of women in Kenya are overweight or obese. The occurrence of overweight/obese mother−child undernutrition pairs is not an independent nutrition problem but rather a result of increasing prevalence of maternal overweight status in the population^(34)^. This rapid rise in overweight/obesity in the adult population is faster than the decline in childhood undernutrition in Kenya.

We found notable levels of mother−child pairs of double burden of malnutrition in Kenya. The form of double burden prominent in the country was the overweight/obese mother−stunted child pair while the overweight/obese mother−underweight or wasted child occurred to a lesser extent. The overweight/obese mother–stunted phenomenon has been reported in a poor urban slum in Kenya^(6)^ and other parts of sub-Saharan Africa^(35)^. We found that living in urban areas, higher household wealth, and having a male child in the mother−child pair were significant predictors of the overweight/obese mother−stunted child pairs. At the bivariate level of analysis, access to improved water and sanitation facilities, lack of maternal education, younger maternal age, higher maternal parity and older child age, were associated with the overweight/obese mother−stunted child pairs. However, these associations were not statistically significant in the adjusted logistic regression model. Although maternal education is a known determinant of child nutrition outcomes^(9,32,36,37)^, maternal education is positively correlated with household wealth^(38)^ and this correlation may explain the lack of statistical significance of maternal education for the double burden in the adjusted multivariate logistics model. Access to water and sanitation facilities are included in the DHS household wealth quintile variable. When included in the adjusted logistic model as independent variables, the water and sanitation facilities were not statistically significant determinants of the double burden.

In our present analysis, we found the proportion of overweight/obese mothers was equal for both the mothers of boys and the mothers of girls. However, the proportion of stunted boys was higher than that of girls. When the mother−child pair was considered, the overweight/obese–stunted child pairs more likely occurred among the mother−boy pairs than the mother–girl pairs. Our findings that the overweight/obese mother−stunted child pairs were more likely to occur if the child was male is not surprising noting that the sex differential in stunting rates has been consistently higher in boys than girls in Kenya^(9)^ and in similar settings^(39–41)^. The sex differential in stunting draws various biological and behavioural explanations. Biological explanations include the chromosomal differences between males and females that depict morbidity and mortality to be higher in male children than females^(41,42)^. Behavioural speculations about the sex differentials in stunting include aspects of selection bias, parental preferences of one sex over another, and cultural perceptions^(41)^. For instance, in Guatemala male infants have been reported as receiving complementary feeding younger than 6 months compared with girls because of cultural perceptions that infant boys were hungrier than girls and breastfeeding alone was not adequate for the boys^(43)^. Early introduction to complementary feeding compromises the infant's immunity due to potential exposure to pathogens and increases the risk of infections^(42)^. Early complementary feeding reduces production of breast milk in the mother^(42)^ and may consequently results in more hungry children, increasing the need for more complementary foods. Breastfeeding is a vital component of the infant and young child feeding practices that promote healthy growth and development of children and reduces the incidence of undernutrition^(44–46)^. Poor breastfeeding patterns and suboptimal complementary feeding practices are widespread in low- and middle-income countries^(47)^. Suboptimal child feeding practices are associated with growth faltering and poor nutrition outcomes of young children^(48–50)^.

In this study, the levels of child stunting and underweight were higher in rural areas while the prevalence of maternal overweight and obesity was higher in the urban areas (Supplementary Table S1). However, overweight/obese mother−stunted child pairs were more likely to occur in urban residence and higher wealth quintile households. Similar to our findings, Jehn & Brewis reported that living in urban households increased the likelihood of mother overnutrition−child undernutrition pairs^(51)^. Our findings are an indication that maternal overweight in urban areas in Kenya is an important contributor to the urban–rural differentials of the double burden. Although childhood undernutrition is reducing in the country, maternal overnutrition and the double burden undermines the gains the country has made in the fight against undernutrition. In affluent urban areas, higher incomes encourage adoption of a more sedentary lifestyle which reduces physical activity, consequently lowering energy expenditure and resulting in overweight and obesity in adults^(52)^. Household wealth in the DHS is measured using a composite variable based on a list of household possessions such as type of building materials for the house, access to clean drinking water and sanitation facilities, ownership of a car, electronics such as television, radio, refrigerator and telephones. Car ownership provides a more sedentary commute to work rather than on public transport that may encourage some walking to access a bus or train ride. Watching television is described as a proxy for seating time and was associated with overweight and obesity in Bangladesh^(42)^. A shift from manual labour to mechanised operations, from walking to motorised transportation, from physically labour-intensive economic activities to desk-bound work reduces energy expenditure. Sedentary lifestyle such as routinely sitting in a computer-based typical office chair is positively correlated with body weight^(53)^. Our study did not show any differences in the double burden of malnutrition between working and not working mothers. However, the work status as measured in this study included all forms of work and did not separate formal and informal types of work. In urban Kenyan cities such as Nairobi, it is common practice for working mothers in the formal sector to employ full-time domestic house helps who assist in household chores including child care, cleaning and meal preparation^(54)^. Employing full-time domestic workers potentially compromises the quality of care that the child receives while the mother is out of home and away at work^(55)^. In addition, several studies have reported maternal employment as a persistent barrier to breastfeeding^(56–60)^.

The nutrition transition is associated with the emergence of a malnutrition double burden^(2,51)^. Although our analysis did not look at dietary intake variables, there is evidence of a nutrition transition in Kenya and Tanzania^(61)^, which is characterised by a shift in dietary patterns from traditional foods to the consumption of energy-dense and nutrient-poor foods. The nutrition transition is propagated by a dietary shift from plant-based high-fibre diets to processed high-energy-dense foods^(62)^. The energy-dense foods lacking in other essential nutrients offer suboptimal nutrition for the children while contributing to weight gain in the mothers, thus the resultant double burden of malnutrition.

Our study had some strengths and limitations. Strengths include the use of nationally representative data to examine the double burden of maternal and child pairs that has not been reported previously at national scale. Although the analysis included a large sample of children and mothers, the sub-analysis of the double burden for child underweight and wasting indicators was limited due to reduced underweight and wasting levels in the country. This analysis does not include dietary intake and physical activities which are important determinants of the double burden because such data are not included in the DHS. The cross-sectional nature of the data did not allow for an analysis of causal relationships for the double burden.

### Conclusion

The double burden of overweight/obese mother−stunted child pairs is present in Kenya. The double burden dyads are more likely to occur in urban areas and in richer wealth quintile households. It is noteworthy that the boys have higher levels of stunting than girls and the overweight/obese mother−stunted child pairs are more likely to occur in the pairs with boys than those with girls. This phenomenon calls for further research to explore if there exist specific determinants of the mother−boy child dyad double burden. The double burden of malnutrition in the country presents a novel public health problem that complicates nutrition policy planning and interventions. This evidence is important to inform a policy and programming shift to address the mother−child dyads rather than attending to child undernutrition separate form maternal overnutrition.

## Availability of data and material

The datasets analysed in this study are publicly available at the DHS Program website^(25)^.

## Supplementary material

For supplementary material accompanying this paper visit https://doi.org/10.1017/jns.2019.39. click here to view supplementary material
